# Targeting Hydrogen Sulfide Modulates Dexamethasone-Induced Muscle Atrophy and Microvascular Rarefaction, through Inhibition of NOX4 and Induction of MGF, M2 Macrophages and Endothelial Progenitors

**DOI:** 10.3390/cells11162500

**Published:** 2022-08-11

**Authors:** Mohamed Adel, Hassan Reda Hassan Elsayed, Mohammad El-Nablaway, Shereen Hamed, Amira Eladl, Samah Fouad, Eman Mohamad El Nashar, Mohammed Lafi Al-Otaibi, Mohammed R. Rabei

**Affiliations:** 1 Department of Medical Physiology, Faculty of Medicine, Mansoura University, Mansoura 35516, Egypt; 2 Department of Anatomy and Embryology, Faculty of Medicine, Mansoura University, Mansoura 35516, Egypt; 3 Department of Anatomy, Faculty of Physical therapy, Horus University, New Damietta 34517, Egypt; 4 Department of Medical Biochemistry and Molecular Biology, Faculty of Medicine, Mansoura University, Mansoura 35516, Egypt; 5 Department of Medical Biochemistry, College of Medicine, Almaarefa University, Riyad 71666, Saudi Arabia; 6 Department of Medical Histology and Cell Biology, Faculty of Medicine, Mansoura University, Mansoura 35516, Egypt; 7 Department of Pharmacology, Faculty of Medicine, Mansoura University, Mansoura 35516, Egypt; 8 Medical Experimental Research Center (MERC), Faculty of Medicine, Mansoura University, Mansoura 35516, Egypt; 9 Department of Anatomy, College of Medicine, King Khalid University, Abha 61421, Saudi Arabia; 10 Department of Histology and Cell Biology, Faculty of Medicine, Benha University, Benha 13511, Egypt; 11 Department of Orthopedics, College Medicine, King Khalid University, Abha 61421, Saudi Arabia; 12 Department of Physiology, Faculty of Medicine, King Salman International University, El Tor 46511, Egypt

**Keywords:** muscle atrophy, H2S, NaHS, MGF, NOX-4, Myostatin

## Abstract

Long-term use of Glucocorticoids produces skeletal muscle atrophy and microvascular rarefaction. Hydrogen sulfide (H2S) has a potential role in skeletal muscle regeneration. However, the mechanisms still need to be elucidated. This is the first study to explore the effect of Sodium hydrosulfide (NaHS) H2S donor, against Dexamethasone (Dex)-induced soleus muscle atrophy and microvascular rarefaction and on muscle endothelial progenitors and M2 macrophages. Rats received either; saline, Dex (0.6 mg/Kg/day), Dex + NaHS (5 mg/Kg/day), or Dex + Aminooxyacetic acid (AOAA), a blocker of H2S (10 mg/Kg/day) for two weeks. The soleus muscle was examined for contractile properties. mRNA expression for Myostatin, Mechano-growth factor (MGF) and NADPH oxidase (NOX4), HE staining, and immunohistochemical staining for caspase-3, CD34 (Endothelial progenitor marker), vascular endothelial growth factor (VEGF), CD31 (endothelial marker), and CD163 (M2 macrophage marker) was performed. NaHS could improve the contractile properties and decrease oxidative stress, muscle atrophy, and the expression of NOX4, caspase-3, Myostatin, VEGF, and CD31 and could increase the capillary density and expression of MGF with a significant increase in expression of CD34 and CD163 as compared to Dex group. However, AOAA worsened the studied parameters. Therefore, H2S can be a promising target to attenuate muscle atrophy and microvascular rarefaction.

## 1. Introduction

The long-term use of Glucocorticoids, widely-used anti-inflammatory agents, causes many adverse effects. One of the most common adverse effects of Dexamethasone, a type of Glucocorticoid, is that it can promote proteolysis and hence muscle atrophy [1], through induction of NADPH (nicotinamide adenine dinucleotide phosphate, reduced form) oxidase NOX4, with a predominant role in mediating oxidative stress in skeletal tissue [2], through inhibition of insulin-like growth factor 1 (IGF-1), a major factor for skeletal muscle growth [3], and through activation of Myostatin which arrests skeletal muscle cell growth [4].

Fast-twitch glycolytic fibers are more susceptible to atrophy than slow-twitch oxidative fibers [5]. However, Dexamethasone (DEX) has been found to cause microvascular rarefaction and micro-vessel loss in slow-twitch muscles as soleus muscle and to significantly reduce the capillary-to-fiber ratio, capillary density, angiogenic factors, vascular endothelial growth factor (VEGF), and its receptor 2 (VEGFR-2) with increased apoptosis as marked by a decreased Bcl2/Bax ratio and increased caspase-3 level [6].

A question was raised concerning whether the muscle atrophy induced by DEX is a direct effect or indirect effect, through the inhibition of skeletal muscle angiogenesis. Langendorf et al. [7] found that DEX inhibited the expression of angiogenic markers and suppressed the VEGF-provoked angiogenic effect of myoblasts; however, interestingly, DEX enhanced the expression of myogenic transcription factors. Moreover, DEX was found to enhance the anti-inflammatory M2 macrophages involved in myogenesis and muscle repair, while repressing the proinflammatory M1 involved in myofiber lysis [8].

Hydrogen sulfide (H2S) is a gasotransmitter that can be endogenously produced by a large number of cells and tissues. H2S influences a wide range of cellular functions, such as cell proliferation and differentiation, oxidative stress, cellular bioenergetics, and metabolism [9]. H2S has been found to have a potential role in skeletal muscle regeneration in health and disease. In broiler chick, a supplement of H2S was able to stimulate breast muscle development by regulating protein synthesis [5]. H2S was found to protect the diaphragm muscle fibrosis and strengthen diaphragmatic biomechanical properties in streptozotocin-induced diabetic rats in another study [10]. H2S was also found to increase muscle mass in db/db diabetic mice [11].

H2S was found to have a potential role in skeletal muscle regeneration in health and disease. However, the mechanisms still need to be elucidated. In this study, we investigated the in vivo role of exogenous H2S donor, NAHS or its inhibitor, in the manipulation of NOX4, apoptosis, Mechano-growth factor (a spliced variant of insulin-like growth factor 1), endothelial progenitors, and M2 macrophage, after muscle injury and microvascular rarefaction, induced by DEX in rats.

## 2. Materials and Methods

### 2.1. Sample Size Estimation

The G*Power tool (Version 3.1.9.2, by Franz Faul, Kiel, Germany) was used to compute the sample size using the procedures given by Faul et al. [12], to limit the number of animals involved. In light of past research articles, we assumed that means, standard deviations, effect sizes (f), and thus the sample sizes for the four groups would be as shown in Table 1, and reach a power of 95% to identify these effect sizes at 5% alpha level, taking into consideration the least effect size (1.0308) and one-way ANOVA plan with the four groups, suggesting a sample size of four/group. Utilizing an F-test, the total number of 24 for the sample achieves a power of 95% to achieve a significance level of 0.05. 

### 2.2. Ethical Statement

The research design used in our study complies with the Animals in Research: Reporting in Vivo Experiments (ARRIVE) criteria and was also approved by the Faculty of Medicine’s Research Ethics Committee (Institutional Research Board), Mansoura University, Egypt (proposal code: R.21.01.1167. R1-2021/02/03).

### 2.3. Research Design

Twenty male adult Sprague–Dawley (SD) rats of wild type (200 ± 40 g in weight, 7–8 weeks in age) were bought from Mansoura University Research Center (MERC), Faculty of Medicine, Mansoura University, Egypt. SD rats were used because they are good quality models for muscle sarcopenia research [16] and males were chosen to avoid the protective effect of estrogens on skeletal muscle [17]. In MERC, the rats were kept in stainless-steel mesh-bottomed cages (three rats per cage) with accurate 12/12 light/dark cycles, temperature, humidity, and aseptic pathogen-free conditions, with free ad libitum access to food and drink. The rats were allowed to acclimatize through the housing for two weeks before the beginning of the study. To avoid bias, the rats were placed in a random manner into four groups: Group 1: negative control group (*n* = 6), in which the rats received saline (Sal. group); Group 2: DEX group (*n* = 6), in which the rats received a subcutaneous injection of with Dexamethasone (DEX) (Amriya, for pharmaceutical industries, Egypt) (0.6 mg/Kg/day) for six days per week for two weeks [18]; Group 3: DEX + NaHS group (*n* = 6), in which the rats received DEX as mentioned in Group 2, in addition to an intraperitoneal injection of NaHS (the donor of H2S) (Acros organics © Belgium), dissolved in saline (5 mg/Kg/day) for two weeks [19]; and Group 4: DEX + AOAA (*n* = 6), in which the rats received DEX as mentioned in Group 2. In addition, they received intraperitoneal injection of Aminooxyacetic acid (AOAA), a blocker of H2S (Acros organics © Belgium) (10 mg/Kg/day) for two weeks. AOAA was used as it can inhibit the two major sources for endogenous enzymatic production of H_2_S, which are cystathionine β synthase (CBS) and cystathionine γ lyase (CSE), rather than the other inhibitors [20]. At 11 a.m., all interventions were completed. The rats were anesthetized with ether inhalation and subsequently slaughtered by cervical dislocation after 14 days of testing.

### 2.4. Sample Preparation

The soleus (SOL) muscle of the left leg was dissected from the rats and rapidly excised, washed with saline, and then dried on a filter paper. NOX4, Myostatin, and Mechano-growth factor (MGF) gene expression levels were measured in a portion of each muscle that was promptly frozen in liquid nitrogen at −80 °C. Meanwhile, the rest of the soleus muscle was processed for histopathological investigation.

### 2.5. Muscle Functional Study

#### 2.5.1. Muscle Force Measurements

A femur transection was performed to remove the right leg. The leg was then immediately placed in a Krebs buffer in a polycarbonate chamber at pH 7.4, constant 95 percent O_2_ and 5 percent CO_2_, and kept at 35 degrees Celsius. To reduce excessive muscular damage, the origin of the SOL muscle was preserved. The leg is horizontally positioned and fastened to the chamber bottom with metal hooks. Biopac Student Lab (Biopac Systems Inc., Goleta, CA, USA) was used to carry out direct electrical stimulations with a stimulator (the Biopac student isolation stimulator module; includes AC 100A power), force transducer assembly (SS121LA; includes a S hook), and tension adjuster (HDW 100A). The muscle was placed at a 90° angle to the leg when the force transducer arm was placed. The muscles were positioned at the ideal length for maximum isometric twitch force measurement in all studies. Field stimulations through two platinum plate electrodes applied supra-maximal currents of 0.2 ms duration was used to record muscle contractions. Micro-manipulations of muscle length and a series of twitch contractions (1 Hz square wave pulse) were performed to obtain the best muscle length and voltage of supra-maximal stimulation. The muscle was then rested for at least 30 s between twitch responses until twitch tension reached maximal. The optimal muscle length (Lo) is measured using a digital caliper and is defined as the length required to deliver the maximum twitch force. Supra-maximal stimulation is the smallest amount of current required to make sure that all muscle fibers are recruited with a single action potential. At the end of the muscular stimulations, the isometric muscle contraction was recorded in g tension. The time it took to attain peak twitch tension and the time it took to reach 50% relaxation were used to determine the contractile characteristics’ speed [21].

#### 2.5.2. Establish Frequency–Force Relationship

After achieving Lo, the frequency–force relationship may be determined by stimulating the muscle with grading frequencies of 10, 30, 50, 80, 100, 120, 150, 180, 200 and 250 Hz. Between stimuli, the muscle was rested for 3–5 min, and stimulation was administered for 500–900 milliseconds. The greatest absolute isometric tetanic force was calculated using the frequency–force relationship’s plateau (Po). A total of 100 Hz was used to obtain Po for the soleus muscle. The muscle was then held in an organ bath for five minutes to recover from fatigue after tetanic stimulation before being stimulated with a single supra-maximal stimulus to record the strength of muscular contraction after recovery [21].

#### 2.5.3. Maximum Isometric Tetanic Force

Muscles were typically stimulated 2–3 times with 3–5-min rest periods to identify the optimum force generation using the supra-maximal voltage at Lo and a plateau stimulation frequency [21].

#### 2.5.4. Specific Force Calculations

Because absolute Po is dependent on muscle size, Po measurements were normalized for the cross-sectional area. Specific force (sPo) in gm/m^2^ was obtained by dividing Po by the calculated total muscle cross-sectional area [21].

### 2.6. Measurement of Muscle Weight and Cross-Sectional Area

The muscles were separated and the tendons of both the proximal and distal ends were severed after the muscle contraction was recorded. The muscles were then dried twice using wipes before being weighed. The following equation was used to compute the muscle cross-sectional area: cross-sectional area = (Muscle mass, in gram)/[1.06 g/cm^3^ × (optimal fiber length, in cm)]. The muscle density is 1.06 g/cm^3^, and the muscle density is 1.06 g/cm^3^. Also, 0.6 or 0.71 × Lo was calculated as the ideal fiber length. The ratio of fiber length to the Lo of the soleus muscle is 0.71 [22].

### 2.7. Serum K⁺ and Creatine Kinase-MM (CK-MM) Assay

Blood samples were obtained from the heart in EDTA-free tubes at the end of the study. Blood was allowed to coagulate at room temperature before being centrifuged for 15 min at 3000 rpm (Hettich universal 32A, Germany) to extract serum. The serum samples were kept in aliquots at −20 °C until they were analyzed. K^+^ and CK-MM levels in the samples were determined using commercially available kits.; potassium kit (Bio-diagnostic, Egypt, cat.No.PT 1820), CK-MM sensitive ELISA kit purchased from (Abnova, Taiwan, cat. No. KA2072) All assays were performed in accordance with manufacturer’s guidelines [23].

### 2.8. Total Antioxidant Capacity (TAC)

The Trolox equivalent antioxidant capacity (TAC) assay was used to determine total antioxidant capacity. Trichloroacetic acid was used to deproteinize the samples. The radical solution had an absorbance of 0.70.02. Then, using a UV spectrophotometer (T80 + UV/VIS Spectrometer PG Instruments Ltd., Lutterworth, UK), the decrease in absorbance caused by antioxidant capacity in the sample was determined at 734 nm and compared to that of Trolox standards [24].

### 2.9. Assay of Lipid Peroxidation Marker Malondialdehyde (MDA) and Antioxidant Reduced Glutathione (GSH) Activity in Muscle Tissues

Applying a mortar and pestle, approximately 50–100 mg of muscle tissues were homogenized in 1–2 mL cold buffer (50 mM potassium phosphate, PH 7.5, 1 mM EDTA), then centrifuged at 4000 rpm for 15 min at 4 °C. The supernatant was stored at −20 °C until oxidants and antioxidants were measured. Then we determined the concentrations of MDA and GSH in the supernatant by a colorimetric technique, as recommended by the manufacturer (Bio-Diagnostics, Dokki, Giza, Egypt). 

### 2.10. RNA Isolation and RT-PCR

The QIAzol reagent (Qiagen, Germany) was used to extract total cellular RNA according to manufacturer guidelines. The NanoDrop 2000 from ThermoScientific (USA) was used to determine RNA concentration. 1 μg of RNA was reverse transcribed using the Bioline cDNA synthesis kit (Bioline, Taunton, MA, USA).

Real-time PCR equipment (Pikoreal 96, ThermoScientific) was used to replicate cDNA templates. The amplification process consisted of a 20 μL total volume mixture [10] μL of HERA SYBR green PCR Master Mix (Willowfort, West Midlands, UK), 2 μL of cDNA template, 2 μL (10 pmol/μL) of each gene primer, and 6 μL of nuclease-free water], and was carried out using the following program: 95 °C for 2 min, followed by 40 cycles of 95 °C for 10 s, and 60 °C for 30 s. Glyceraldehyde-3-phosphate dehydrogenase (GAPDH) was employed as a control gene [25], and the sequences of the used primer pairs are listed in Table 2. Vivantis provided the primer sets (Vivantis Technologies, Malaysia). Relative gene expression levels were represented as ΔCt = Ct target gene − Ct control gene; fold change of gene expression was calculated according to the 2^−ΔΔCT^ method [26].

### 2.11. Histopathological Examination of Muscle Tissue 

Parts of the soleus muscles in the left leg were immersed in 10% neutral formalin and then embedded in paraffin blocks. A total of 5 μm thick sections of paraffin blocks were cut and processed for routine hematoxylin and eosin staining [27]. To determine capillary density, capillaries were counted under a light microscope to determine the capillary density/high power field (×400). 

### 2.12. Immunohistochemical Study

Sections of 3 μm thickness were used following the immunoperoxidase technique mentioned in Elsayed et al. [28]. In brief, the slides were deparaffinized and endogenous peroxidase was blocked. We added hydrogen peroxide and 0.3% methanol to the liver sections at room temperature for 10 min. Then the soleus muscle sections were heated for 10 min at 95 °C in 10 mM citrate buffer to induce antigen retrieval, then the sections were left to cool for one hour. The sections were then incubated with the primary antibodies for caspase-3 (apoptosis marker), CD34 (a marker of vascular endothelial progenitor cells), VEGF, CD31 (Endothelial marker), and CD163 (M2 anti-inflammatory macrophage marker) overnight at 4c. Table 3 introduces the details of the antibodies and their dilutions. The slides were then incubated with a secondary anti-mouse antibody (sc-516102, Santa Cruz) for 30 min. After, DAB was added for 4 min, the sections were then counterstained with hematoxylin. PBS was added to substitute for the primary antibody as a reagent (no primary antibody) control. Lastly, washing, dehydration, and examination of the slides by light microscope were carried out. Dark brown areas, on a blue background, demonstrate positive staining. Antigen localization was cytoplasmic and nuclear for caspase-3, cytoplasmic for the expression of CD34 and VEGF, and cytoplasmic and membranous for CD31 and CD163.

### 2.13. Morphometric Analysis

Morphometric analysis of the immunohistochemical study was performed using ImageJ software [29] and Fiji ImageJ software [30] (ImageJ 1.52p, by Wayne Rashband, National Institute of Health, Bethesda, MD, USA). We counted the number of immunopositive cells in simple random non-overlapping fields (×400) per group.

### 2.14. Statistical Analysis

Data were entered and analyzed using SPSS (IBM Corp. Released 2017. IBM SPSS Statistics for Windows, Version 25.0. Armonk, NY, USA: IBM Corp.). The Shapiro–Wilk test was used to verify normality in quantitative data, with *p* > 0.050 indicating that the data were normally distributed. Boxplots were used to detect the existence of noticeable outliers. Normally distributed quantitative data were expressed as mean ± standard deviation (SD). One-way ANOVA was used to compare quantitative data between the study groups. To make a proper comparison among the data, Tukey post-hoc adjustment was employed when the assumption of equal variances was assumed. The results were considered significant when the *p* value was ≤0.050. 

## 3. Results

### 3.1. Changes in Soleus Muscle Mass, Length and Cross-Sectional Area 

A significant difference in muscle mass, length, and cross-sectional area of soleus muscle was observed among the studied groups (*p* < 0.0005). When compared to the negative control, the Dex and Dex + AOAA groups had significantly lower muscular mass, length, and cross-sectional area of the soleus muscle. Moreover, no significant difference in these parameters was observed between the Dex + AOAA group and Dex group. Furthermore, the administration of NaHS improved these parameters as compared to the Dex group and demonstrated no significant difference from the negative control group (Table 4).

### 3.2. Changes in Isometric Contractile Properties of Soleus Muscle

**Figure 1:**
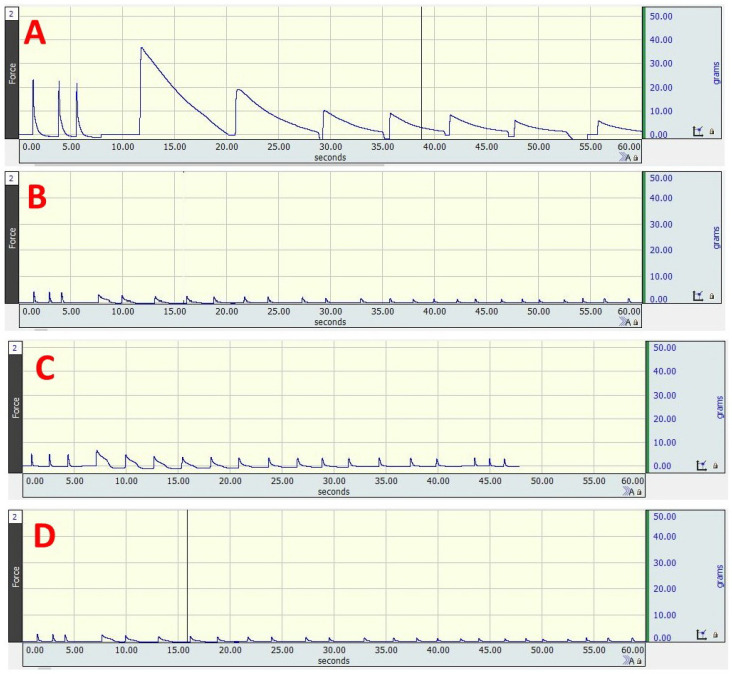
Isometric contractile properties of the soleus muscle in different experimental groups. (**A**) Control. (**B**) Dex. (**C**) Dex + H2S donor (NaHS). (**D**) Dex + H2S blocker (AOAA). Contractility recording was achieved by using Biopac Systems Inc. (BSL 3.7.5 software), (MP36) data analysis unit, Biopac force transducer assembly (SS12LA), Biopac BSLSTM stimulator, and needle electrodes (ELSTM2).

A significant difference in muscle contractile parameters among the studied groups was observed (*p* < 0.0005). When compared to the negative control, a dramatic decrease in maximum isometric twitch force, tetanic force, specific force, and force following tetanic contraction, as well as a significant elongation in time to maximum twitch and half relaxation time were observed in the Dex group and Dex + AOAA group. Moreover, no significant difference in these parameters was observed between the Dex + AOAA group and Dex group. Supplementation of NaHS improved these parameters as compared to the Dex group. Dex + NaHS showed no significant difference from the negative control group except for a significantly higher time to peak twitch (Table 5 and Figure 1).

### 3.3. Oxidative Stress Markers, Serum K⁺ Level, and Creatine Kinase-MM (CK-MM)

A significant difference in the studied parameters was observed among the studied groups (*p* < 0.0005). A significant elevation in MDA, NOX-4, and CK-MM together with a reduction in K, GSH, and TAC were observed in Dex group and Dex + AOAA group as compared to the negative control group. Moreover, no significant difference in these parameters was observed between the Dex + AOAA group and the Dex group except for K and CK-MM. These markers showed significant improvement by NaHS administration as compared to the Dex group with no significant difference from the negative control group except in GSH which was still significantly different from the negative control group (Table 6).

### 3.4. mRNA Expression of Myostatin, MGF, and NOX-4 in Soleus Muscle Tissues

**Figure 2:**
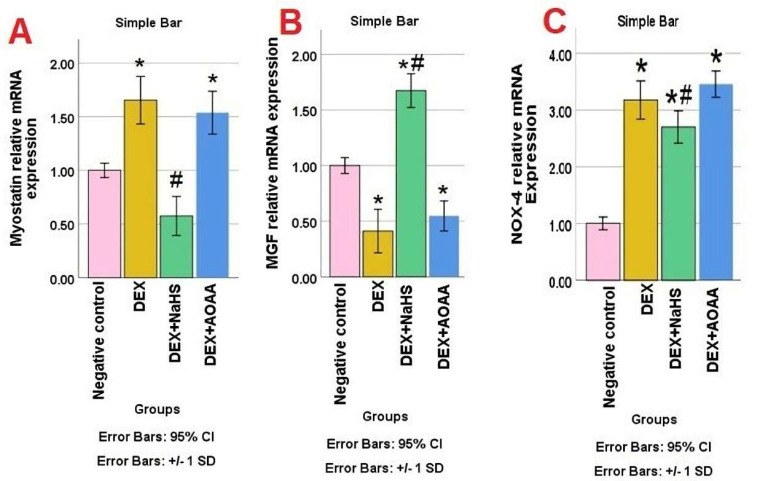
Expression levels of Myostatin (**A**), MGF (**B**), and NOX-4 (**C**) in all studied groups. Data are expressed as mean ± SD, the test used: one-way ANOVA, followed by post-hoc Tukey test (normalized by GAPDH, products of RT-PCR). Dex: Dexamethasone, NaHS: Sodium hydrosulfide and AOAA: Aminooxyacetic acid. *p*: significance (<0.05). *: significance as compared to the control (**C**) group. #: significance as compared to (Dex) group.

A statistically significant difference in Myostatin mRNA expression was observed among the studied groups (*p* value: <0.0001, F value: 47.482). A significant increase in Myostatin expression was observed in the Dex group and DEX + AOAA group as compared to the negative control group. DEX + AOAA showed no significant difference as compared to the DEX group. Muscles from Dex + NaHS rats showed a significant decrease in Myostatin expression as compared to the DEX group and showed no significant difference as compared to the negative control (Figure 2A). A statistically significant difference was observed in MGF mRNA expression among the studied groups (*p* value: <0.0001, F value: 91.987). A significant decrease in MGF expression was observed in the Dex group and DEX + AOAA group as compared to the negative control. DEX + AOAA showed no significant difference as compared to the DEX group, while muscles from Dex + NaHS rats show a significant increase in MGF expression as compared to the Dex group, and negative control (Figure 2B). A statistically significant difference in NOX-4 mRNA expression was observed among the studied groups (*p* value: <0.0001, F value: 111.148). NOX-4 mRNA expression increased significantly in the Dex group and DEX + AOAA group as compared to the control group. DEX + AOAA showed no significant difference as compared to the DEX group. NaHS treatment attenuated the increase in mRNA expression of NOX-4 as compared to the Dex group but was still significantly higher than the negative control group (Figure 2C). 

### 3.5. Results of Haematoxylin and Eosin (HE)- Stained Sections

**Figure 3:**
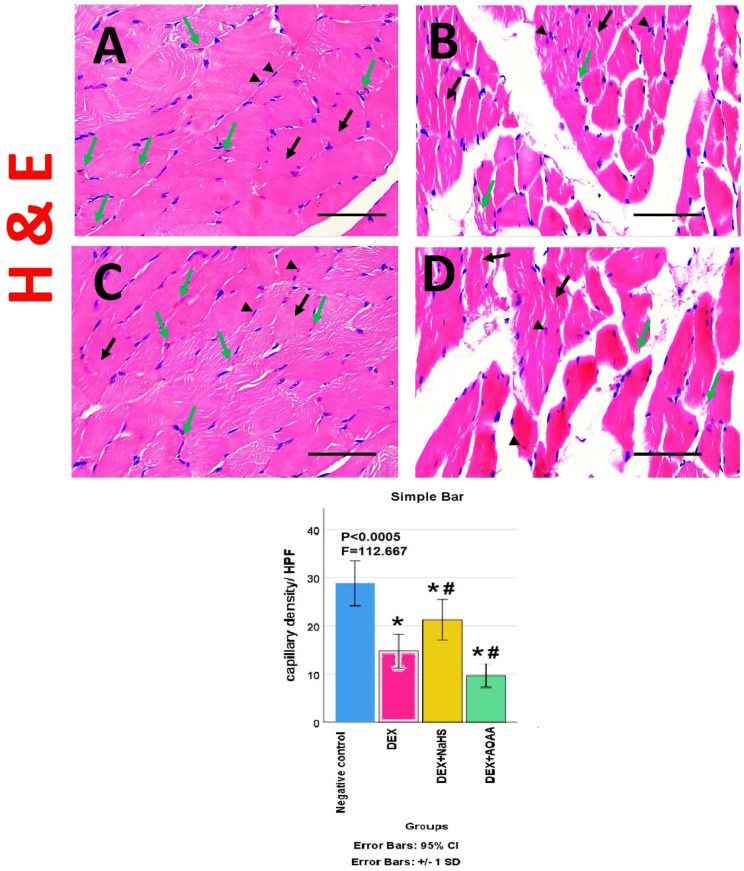
(**A**–**D**) Photomicrographs of transverse sections for the soleus muscle of different groups: (**A**) Group I (negative control) showed normal appearance of skeletal muscle fibers and closely-packed bundles with polygonal acidophilic muscle fibers (black arrows) with peripheral oval nuclei (arrow heads) and no atrophic changes with a big number of capillaries (green arrows); (**B**) Group II (Dex) shows loosely-packed muscle bundles, many fibers with fragmented cytoplasm (black arrows) and displacement of some nuclei away from the periphery (arrows heads), as well as bundle and fiber atrophy, angling and/or degeneration with a few number of capillaries (green arrows); (**C**) Group III (Dex + NaHS) shows remarkable preservation of the normal structure with closely-packed muscle bundles, muscle fibers with polygonal acidophilic muscle fibers no remarkable atrophic changes (black arrows) with peripheral oval nuclei (arrowheads). with a big number of capillaries (green arrows); (**D**) group IV (Dex + AOAA) showing loss of normal architecture, loosely-packed muscle bundles and fragmented muscle fibers (black arrows) and fiber atrophy, angling, and/or degeneration, with fragmented cytoplasm and displacement of some nuclei away from the periphery (arrowheads) with a few number of capillaries (green arrows). Scale bar: 25 μm (HE stain × 400). The histogram shows the capillary density/High power field. Dex: Dexamethasone, NaHS: Sodium hydrosulfide and AOAA: Aminooxyacetic acid. HPF: High power field. *p*: significance (<0.05). *: significance as compared to the control (**C**) group. #: significance as compared to (Dex) group.

The HE stained transverse sections (TS) of soleus muscle from the negative control group, showed a normal appearance of skeletal muscle fibers and closely-packed bundles with polygonal acidophilic muscle fibers, peripheral oval nuclei, and no atrophic changes (Figure 3A). While TS of soleus muscle from Dex group showed bundle and fiber atrophy, angling, and/or degeneration, with fragmented cytoplasm and displacement of some nuclei away from the periphery (Figure 3B). Moreover, TS of the Dex + NaHS group showed remarkable preservation of the normal structure with multiple muscle fibers with polygonal acidophilic muscle fibers with peripheral oval nuclei and no remarkable atrophic changes (Figure 3C). Furthermore, TS of soleus muscles from Dex + AOAA group showed loss of normal architecture and fragmented muscle fibers and fiber atrophy, angling, and/or degeneration, with fragmented cytoplasm and displacement of some nuclei away from the periphery (Figure 3D). The capillary density showed a significant decrease in Dex and Dex + AOAA groups as compared to the negative control group and showed a significant increase in the Dex + NaHS group as compared to the Dex group and negative control group (Figure 3E).

### 3.6. Immunohistochemical Results

**Figure 4:**
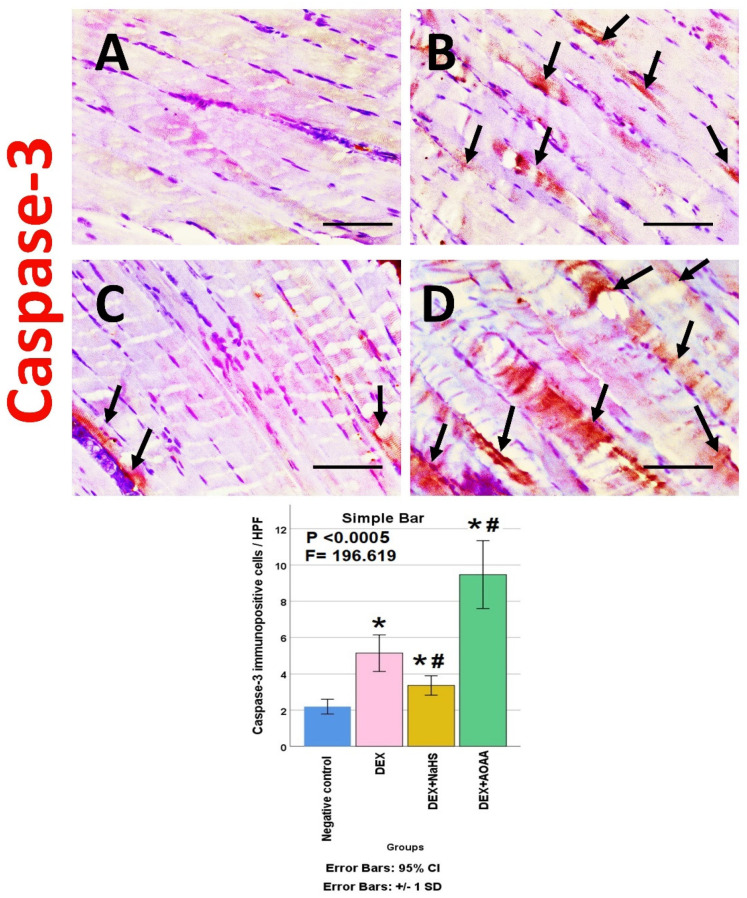
(**A**–**D**): Light-microscopic image of caspase-3 immunostaining (×400) in soleus muscle tissues of all studies groups; Negative control, Dex, Dex + NaHS, and Dex + AOAA groups (**A**–**D**; respectively). Scale bar = 25 µm. Arrows: Caspase-3 immunopositive cells. (**E**) The mean of the number of caspase-3 immunopositive cells/HPF in the studied groups. Results are mentioned as mean ± standard deviation. The test used: one-way ANOVA, followed by post-hoc Tukey test. *p*: significance (<0.05). *: significance as compared to the negative control group. #: significance as compared to (Dex) group. Dex: Dexamethasone, NaHS: Sodium hydrosulfide, and AOAA: Aminooxyacetic acid.

**Figure 5:**
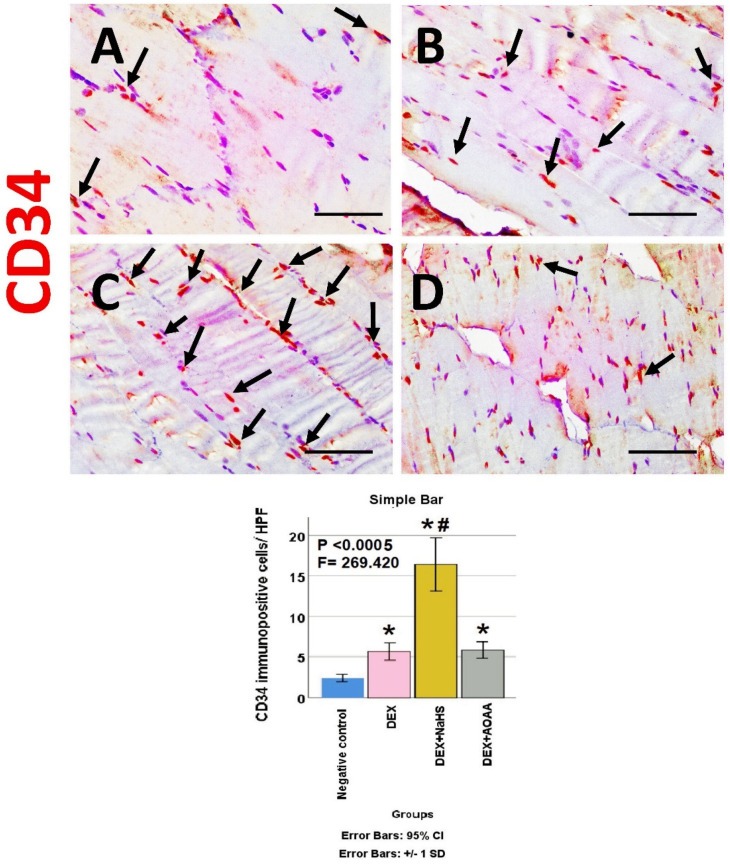
(**A**–**D**): Light-microscopic image of CD34 immunopositive staining (×400) in soleus muscle tissues of all experimental groups; Negative control, Dex, Dex + NaHS, and Dex + AOAA groups (**A**–**D**; respectively). Scale bar = 25 µm. Arrows: CD34 immunopositive cells. (**E**) Morphometric analysis of the mean of the number of CD34 immunopositive cells/HPF in the studied groups. Results are mentioned as mean ± standard deviation. The test used: one-way ANOVA, followed by post-hoc Tukey test. *p*: significance (<0.05). *: significance as compared to the negative control group. #: significance as compared to (Dex) group. Dex: Dexamethasone, NaHS: Sodium hydrosulfide, and AOAA: Aminooxyacetic acid.

**Figure 6:**
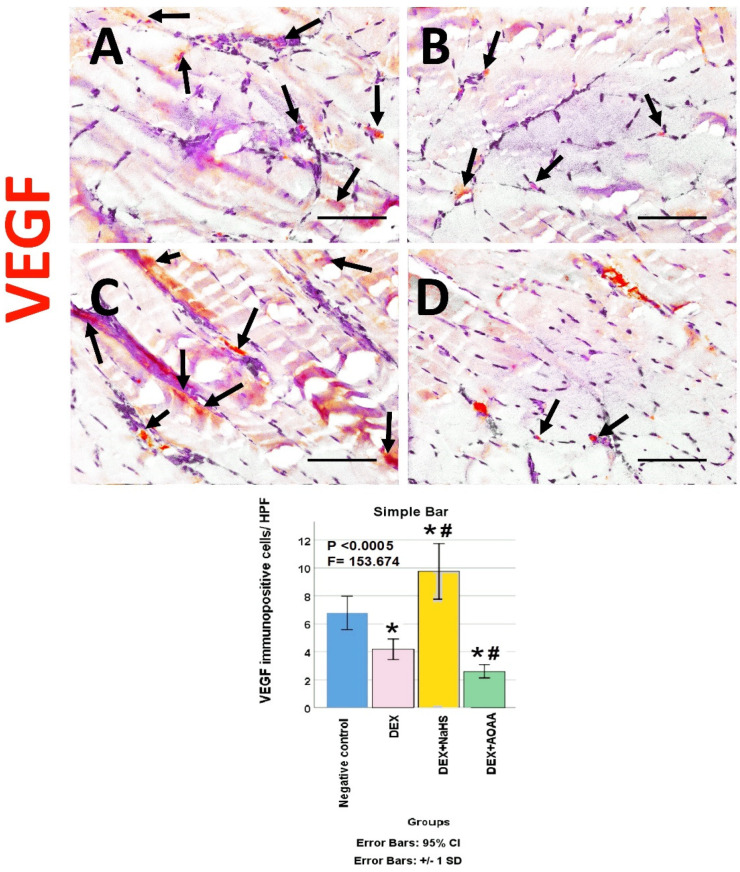
(**A**–**D**): Light-microscopic image of VEGF immunostaining (×400) in soleus muscle tissues of all studied groups; Negative control, Dex, Dex + NaHS, and Dex + AOAA groups (**A**–**D**; respectively). Scale bar = 25 µm. Arrows: VEGF immunopositive cells. (**E**) The mean of the number of VEGF immunopositive cells/HPF in the studied groups. Results are mentioned as mean ± standard deviation. The test used: one-way ANOVA, followed by post-hoc Tukey test. *p*: significance (<0.05). *: significance as compared to the negative control group. #: significance as compared to (Dex) group. Dex: Dexamethasone, NaHS: Sodium hydrosulfide, and AOAA: Aminooxyacetic acid.

**Figure 7:**
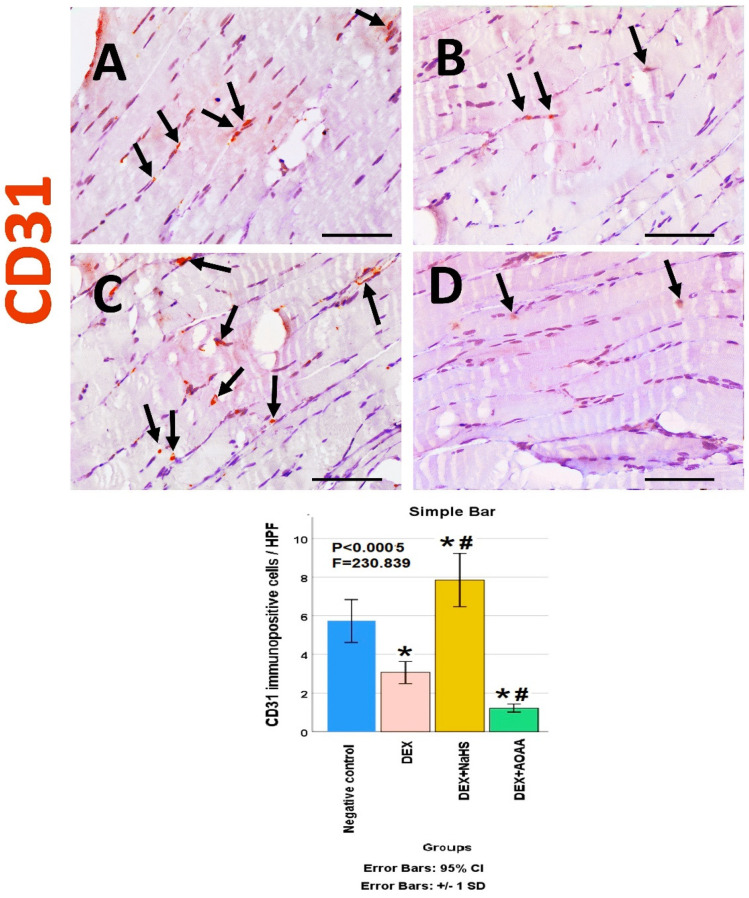
(**A**−**D**): Light-microscopic image of CD31 immunostaining (×400) in soleus muscle tissues of all studied groups; Negative control, Dex, Dex + NaHS, and Dex + AOAA groups (**A**−**D**; respectively). Scale bar = 25 µm. Arrows: CD31 immunopositive cells. (E) The mean of the number of CD31 immunopositive cells/HPF in the studied groups. Results are mentioned as mean ± standard deviation. The test used: one-way ANOVA, followed by post-hoc Tukey test. *p*: significance (<0.05). *: significance as compared to the negative control group. #: significance as compared to (Dex) group. Dex: Dexamethasone, NaHS: Sodium hydrosulfide, and AOAA: Aminooxyacetic acid.

**Figure 8:**
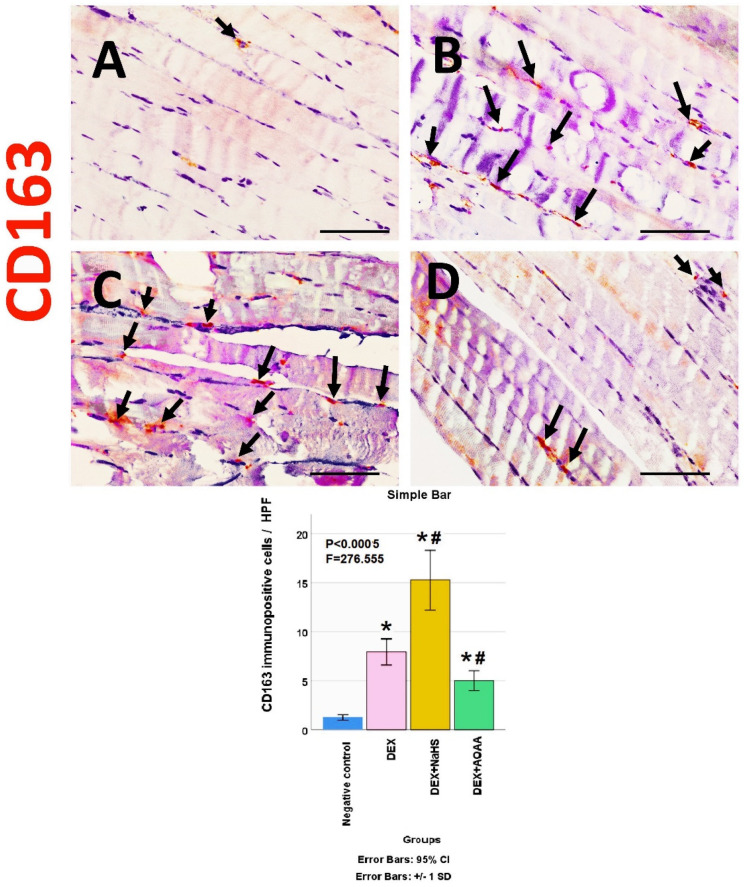
(**A**–**D**): Light-microscopic image of CD163 immunostaining (×400) in soleus muscle tissues of all studied groups; Negative control, Dex, Dex + NaHS, and Dex + AOAA groups (**A**–**D**; respectively). Scale bar = 25 µm. Arrows: CD163 immunopositive cells. (E) The mean of the number of CD163 immunopositive cells/HPF in the studied groups. Results are mentioned as mean ± standard deviation. The test used: one-way ANOVA, followed by post-hoc Tukey test. *p*: significance (<0.05). *: significance as compared to the negative control group. #: significance as compared to (Dex) group. Dex: Dexamethasone, NaHS: Sodium hydrosulfide, and AOAA: Aminooxyacetic acid.

The soleus muscle of the negative control group showed a weak immunoreactivity to caspase-3, CD34, VEGF, CD31, and CD163. Noticeably, muscles from DEX and DEX + AOAA showed an intense expression for caspase-3 with moderate expression of CD34 and CD163, with weak expression of VEGF and CD31. In contrast, DEX + NaHS showed a weak expression for caspase-3 and an intense expression for CD34, CD31, VEGF, and CD163 (Figure 4, Figure 5, Figure 6, Figure 7 and Figure 8). 

### 3.7. Results of Morphometric Analysis of Immunohistochemical Studies

The mean for the number of caspase-3, CD34, VEGF, CD31, and CD163 positive cells/high power field (HPF) revealed a statistically significant difference (*p* < 0.0005) among the studied groups. Post-hoc tests showed that DEX and DEX + AOAA groups showed a significant increase in caspase-3, CD34, and CD163 as well as a significant decrease in VEGF and CD31 expressions as compared to the negative control group. DEX + AOAA group showed a significant elevation in caspase-3 and a significant decrease in CD163, CD31, and VEGF with no significant difference in CD34 expressions as compared to the DEX group. In contrast, the DEX + NaHS group showed a significant increase in CD34, VEGF, CD31 and CD163 expressions as compared to the DEX and the negative control group with a significant decrease in caspase-3 as compared to the Dex group and a significant increase as compared to the negative control group (Figure 4, Figure 5, Figure 6, Figure 7 and Figure 8).

## 4. Discussion

Muscle atrophy is one of the major side effects of DEX [1], with the induction of NOX4 and apoptosis after muscle injury and microvascular rarefaction. Hydrogen sulfide (H2S) a gasotransmitter that can be endogenously produced by different tissues, including skeletal muscles, was found to have a potential role in skeletal muscle regeneration in health and disease [5,10,11]. However, the mechanisms still need to be elucidated. In this study, we investigated the in vivo role of exogenous H2S donor, NAHS, or its inhibitor Aminooxyacetic acid (AOAA), in the manipulation of angiogenesis and M2 macrophage, NOX4, and apoptosis after muscle injury and microvascular rarefaction, induced by DEX in rats.

Fast-twitch glycolytic fibers are more susceptible to atrophy than slow-twitch oxidative fibers [5]. However, in the present study, DEX was found to cause atrophy in the slow-twitch soleus muscle. We found that the cross-sectional area decreased significantly, in agreement with previous data [31,32,33,34]. However, Baehr et al. [35] reported different results. They found no atrophy in Sol after 14 days of DEX treatment in female mice. However, in the latter study, DEX was provided in the drinking water. The different routes of administration of DEX (subcutaneous injection in this study) could explain the somewhat different extent of atrophy. Moreover, we found that DEX could activate Myostatin which arrests skeletal muscle cell growth similar to the findings of Canepari et al. [34]. We also found that DEX suppressed contractile force, similar to the finding of Yamada et al. [36] and Dunlap et al. [37].

In the present study, DEX induced oxidative stress, increased MDA, and decreased GSH with the induction of NADPH (nicotinamide adenine dinucleotide phosphate, reduced form) oxidase NOX4, which has a predominant role in inducing oxidative stress in skeletal tissue, similar to the findings of Bai et al. [2]. NOX4 activity showed higher values in the slow-twitch oxidative fibers as SOL muscle compared to other muscles [38,39]. DEX inhibited Mechano-growth factor (MGF), a unique, spliced variant of insulin-like growth factor 1 (IGF-1), responsible for muscle regeneration and repair after a stretch or mechanical damage. This is consistent with the finding of Canepari et al. [34], who reported a decreased activity of IGF-1; a major factor for skeletal muscle growth, and according to Mostafa and Samir [40] who reported a decreased activity of mechano-growth factor (MGF) with DEX administration.

In the present study, DEX caused microvascular rarefaction and micro-vessel loss with suppression of expression of angiogenic factors; VEGF and endothelial marker; CD31 with increased apoptosis as marked by increased caspase-3 level, similar to the finding of Jesus et al. [6]. A question was raised concerning whether the muscle atrophy induced by DEX is a direct effect or indirect, through inhibition of skeletal muscle angiogenesis. Langendorf et al. [7] found that DEX inhibited the expression of angiogenic markers and inhibited the VEGF provoked-angiogenic effect of myoblasts; however, interestingly, DEX enhanced the expression of myogenic transcription factors. This is similar to our finding of decreased endothelial marker; CD31 expression in the DEX group coincided with increased M2 macrophage number as seen through increased expression of CD163. This is consistent with Chazaud [8], who reported the finding that the anti-inflammatory M2 macrophages are involved in myogenesis and muscle repair and that the proinflammatory M1 is involved in myofiber lysis.

Regarding the H2S donor, NaHS was found to increase the H2S concentration and to abrogate the inhibitory effects of DEX on protein synthesis but in fast-twitch muscle fibers [5]. On the other hand, we report for the first time that the NaHS could rescue soleus slow-twitch muscles, too, while the H2S inhibitor, AOAA, potentiated the atrophy and NaHS suppressed the Myostatin muscle and apoptosis as seen through caspase-3 expression while AOAA increased them. This is similar to the finding of Bitar et al. [41] who reported that NaHS could decrease the expression of Myostatin in diabetes-induced muscle atrophy and that treatment with a potent H2S donor at an early stage of diabetes is likely to mitigate the development of sarcopenia/frailty and predictably reduces its devastating sequelae of amputation. We also found that NaHS restored the muscle contractile force while AOAA worsened it, similar to previous results reporting that Hydrogen sulfide donors protected against mechanical ventilation-induced atrophy and contractile dysfunction in the rat diaphragm [42].

In the present study, we report for the first time that the administration of NaHS to the rats with DEX-induced muscle damage decreased the expression of NOX4, while AOAA increased it, and this may be one of the underlying mechanisms for the muscle rescuing effect of NaHS. This is consistent with previous findings in other organs and models; endogenous H2S was found to inhibit NOX4-provoked oxidative stress in LPS-induced macrophages [43]. H2S alleviated uranium-induced rat hepatocyte cytotoxicity via inhibiting the Nox4/ROS/p38 MAPK pathway [44]. H2S reduced ischemia and reperfusion injury in neuronal cells of the retina through the modulation of transcription factor NF-kappa B and the reduction in retinal inflammation [45].

In the present study, we report for the first time that the administration of NaHS to the rats with DEX-induced muscle damage increased the activity of Mechano-growth factor (MGF), while AOAA decreased it. MGF is an insulin-like growth factor 1 (IGF-1) spliced variant, responsible for muscle regeneration and repair after a stretch or mechanical damage. It had been reported to be markedly more effective than IGF-I [46].

In addition, we found for the first time that the administration of NaHS to the rats with DEX-induced muscle damage, increased the expression of CD34, while AOAA decreased it. CD34 is a transmembrane glycoprotein expressed on stem cells, progenitor cells, and endothelial cells. This is consistent with the finding that MGF has been shown to stimulate muscle stem cells (satellite cells) to re-enter the cell cycle and proliferate, resulting in new muscle cells to replace injured fibers. A similar role of MGF has been explored in chondrogenesis and the differentiation of mesenchymal stromal cells [46,47]. This is also consistent with the finding by Abdelmonem et al. [48], who reported the potential of H2S in the stimulation of cardiac stem cells and preventing cardiomyocyte loss. 

In the present study, the administration of the H2S donor, NaHS, to the rats with DEX-induced muscle damage, increased the number of endothelial cells associated with induced angiogenesis, while the administration of the H2S inhibitor, AOAA, decreased their number, as seen through the expression of CD31 and VEGF. This is consistent with the finding that MGF could accelerate angiogenesis after anterior cruciate ligament (ACL) injury probably owing to its recruitment of proangiogenesis cells by stromal cell-derived factor 1alpha/CXCR4 axis and stimulation of vascular endothelial growth factor alpha expression level [49]. This is also in accordance with the finding by Cheng and Kishore [50], who reported the important role of H2S in maintaining endothelial function/biology and angiogenic property but in diabetes-induced critical ischemic limb.

The macrophage system has a dual role in skeletal muscle injury and repair; proinflammatory M1 macrophages are involved in myofiber lysis while anti-inflammatory M2 macrophages are involved in myogenesis and muscle repair [8]. In the present study, the administration of the H2S donor, NaHS, to the rats with DEX-induced muscle damage, increased the number of M2 macrophages, while the administration of the H2S inhibitor, AOAA, decreased their number as seen through increased CD163 positive cells. This is similar to the finding by Zhao et al. [51], who studied the effect of NaHS but on another muscle injury model, Gastrocnemius (fast-twitch muscle) contusion. H2S treatment reduced the inflammatory M1 macrophage marker (CD68) and increased the anti-inflammatory M2 macrophage marker CD206.

In accordance with our work on skeletal muscles, a similar study on vascular beds was carried out by di Villa Bianca et al. [52]. They reported an obvious decrease in the expression of CBS and CSE as well as H2S production in mesenteric and carotid arteries from the rats receiving Dexamethasone. Moreover, plasma H2S was strongly decreased in DEX-treated rats. Briefly, the early stage of Dex-induced hypertension is associated with a disturbance in H2S system, so antagonizing this disruption may be effective in managing DEX-induced hypertension.

Moreover, a recent published study by Micheli et al. [53] showed in vitro results that are in accordance with the in vivo results we report. They established a Dex-induced sarcopenia in an in vitro model in C2C12-derived myotubes; they studied the role of exogenous and endogenous H2S sources. The H2S donors, Glucoraphanin, 3-mercaptopyruvate, and L-cysteine avoided the alteration in myotube appearance induced by DEX. Glucoraphanin and 3-mercaptopyruvate but not L-cysteine stopped the Dex-induced apoptosis and oxidative stress. These previous results reinforce the in vivo presented results

## 5. Conclusions

This study found that NaHS could improve the muscle contractile properties and decrease the oxidative stress and expression of NOX4, caspase-3, Myostatin, VEGF, and CD31 as well as increase the expression of MGF and cause a significant increase in the expression of CD34 and CD163 as compared to the Dex group. However, AOAA worsened the studied parameters. Therefore, NaHS is a promising target to attenuate Dex-induced skeletal muscle oxidative stress, atrophy, apoptosis, and microvascular rarefaction and increase angiogenesis.

## 6. Study Limitations

Sprague–Dawley rats were selected because they are good models for muscular sarcopenia research [16] and males were chosen to avoid the protective effect of estrogens on skeletal muscle [17]. So, the controversial results of Dex and H2S on soleus muscle may be due to the difference in species or sex. Moreover, it may be due to the different types of skeletal muscles whether they are slow-twitch or fast-twitch muscles. Additionally, the effect of long-term Dex and NaHS use differs from the effect of short-term use. To achieve a better validation of the results, the experiment should be repeated with several doses of NaHS, various animals and species, different sex, different regimens, different types of skeletal muscles, and even more muscle atrophy models.

## Figures and Tables

**Table 1:** Sample size calculation.

Studied Parameter	Reference	Means	Standard Deviations	Effect Sizes	Sample Sizes
CD31	[2]	35, 60, 70 and 80	4	4.1810	8
NOX4	[6]	1, 1.15, 1.3 and 1.65	1.15	1.6073	12
CD163	[13]	2, 3, 25 and 37	3	4.9575	8
Myostatin	[14]	1, 1.2, 1.2 and 4	0.3	4.1466	8
VEGF	[15]	90, 80, 100 and 100	4	1.0308	24

**Table 2:** The sequence of rat primers used in RT-PCR analysis.

Gene	Sequence
Myostatin	Forward primer: TGCTGTAACCTTCCCAGGACCA Reverse primer: GTGAGGGGGTAGCGACAGCAC
NOX-4	Forward primer: TGTTGGGCCTAGGATTGTGT Reverse primer: CTTCTGTGATCCGCGAAGGT
MGF	Forward primer: GGAGGCTGGAGATGTACTGTGCT Reverse primer: TCCTTTGCAGCTTCCTTTTCTTG
Glyceraldehyde-3-phosphate dehydrogenase (GAPDH)	Forward primer: AGGTCGGTGTGAACGGATTTG Reverse primer: TGTAGACCATGTAGTTGAGGTCA

**Table 3:** The primary antibodies applied for immunohistochemistry.

Name	Cat. Number	Source and Clonality	Dilution
Caspase-3	Servicebio GB11532	Rabbit polyclonal	1/500
CD34	Servicebio GB111693	Rabbit polyclonal	1/500
VEGF	Servicebio GB14165	Mouse monoclonal	1/200
CD31	Dako M0823	Mouse monoclonal	1/200
CD163	Servicebio GB11340-1	Rabbit polyclonal	1/500

**Table 4:** General properties of soleus muscle in all experimental groups.

	Saline Control	Dex	Dex + H2SDonor (NaHS)	Dex + H2S Blocker (AOAA)	F Value	*p* Value
Muscle mass (mg)	127.8 ± 13.02	98.35 ± 7.027 *	122.4 ± 14.40 ^#^	93.58 ± 10.24 *	13.161	<0.0005
Optimal muscle length (mm)	12.56 ± 0.615	10.27 ± 0.603 *	11.72 ± 1.345 ^#^	8.983 ± 0.693 *	19.784	<0.0005
Cross-sectional area (μm^2^)	3497 ± 418.5	2470 ± 438.4 *	3229 ± 520.3 ^#^	2060 ± 205.2 *	15.578	<0.0005

Test used: ANOVA followed by post-hoc Tukey test for multiple comparisons. Values are expressed as means ± S.D (*n* = 6). Dex: Dexamethasone, NaHS: Sodium hydrosulfide and AOAA: Aminooxyacetic acid. *: (*p* < 0.05) significant vs. control group, ^#^: (*p* < 0.05) significant vs. Dex group.

**Table 5:** Isometric contractile properties of the soleus muscle.

	Control Sal.	Dex	Dex + H_2_S Donor (NaHS)	Dex + H_2_S Blocker (AOAA)	F Value	*p* Value
Time to peak twitch (ms)	45.19 ± 07.223	25.54 ± 3.252 *	35.62 ± 4.909 ^#^*	23.64 ± 4.656 *	21.941	<0.0005
Half relaxation time (ms)	53.84 ± 10.61	38.20 ± 4.218 *	51.88 ± 2.940 ^#^	35.41 ± 4.335 *	13.368	<0.0005
Max. isometric twitch force (g)	23.77 ± 4.464	5.28 ± 0.992 *	20.56 ± 3.862 ^#^	3.26 ± 0.611 *	72.376	<0.0005
Specific force (gm/m^2^)	63.96 ± 12.01	34.00 ± 6.385 *	57.45 ± 10.79 ^#^	22.42 ± 0.421 *	28.673	<0.0005
Force after tetanic contraction (g)	5.437 ± 0.819	3.863 ± 0.569 *	5.23 ± 0.78 ^#^	3.00 ± 0.56 *	16.710	<0.0005

Test used: ANOVA followed by post-hoc Tukey test for multiple comparisons. Values are expressed as means ± S.D. (*n* = 6). Dex: Dexamethasone, NaHS: Sodium hydrosulfide and AOAA: Aminooxyacetic acid. *: (*p* < 0.05) significant vs. control group. ^#^: (*p* < 0.05) significant vs. Dex group.

**Table 6:** Oxidative stress markers, serum K⁺ level, and serum creatine kinase-MM (CK-MM) in different experimental groups.

	Negative Control	Dex	Dex + NaHS	Dex + AOAA	F Value	*p* Value
MDA (nmol/mg)	194.8 ± 32.31	345.2 ± 38.36 *	239.6 ± 36.39 ^#^	410.6 ± 93.59 *	18.386	<0.0005
GSH (nmol/mg)	4.931 ± 0.158	3.908 ± 0.299 *	4.619 ± 0.318 ^#^*	3.611 ± 0.098 *	39.864	<0.0005
TAC (nmol Trolox Eq/mL)	1.192 ± 0.189	0.745 ± 0.116 *	1.051 ± 0.232 ^#^	0.544 ± 0.074 *	19.016	<0.0005
Serum K⁺ level (mg/dL)	4.513 ± 0.172	4.071 ± 0.125 *	4.353 ± 0.142 ^#^	3.789 ± 0.157 ^#^*	27.146	<0.0005
Creatine kinase-MM (CK-MM) (ng/mL)	17.09 ± 1.485	27.47 ± 4.392 *	19.67 ± 2.299 ^#^	41.21 ± 7.159 *^#^	36.120	<0.0005

Test used: ANOVA followed by post-hoc Tukey test for multiple comparisons. Values are expressed as means ± S.D. (*n* = 6). Dex: Dexamethasone, NaHS: Sodium hydrosulfide and AOAA: Aminooxyacetic acid. *: (*p* < 0.05) significant vs. control group. ^#^: (*p* < 0.05) significant vs. Dex group.

## Data Availability

All data is contained within the article.

## References

[1] Liu L., Koike H., Ono T., Hayashi S., Kudo F., Kaneda A., Kagechika H., Manabe I., Nakashima T., Oishi Y. (2021). Identification of a KLF5-dependent program and drug development for skeletal muscle atrophy. Proc. Natl. Acad. Sci. USA.

[2] Bai S.C., Xu Q., Li H., Qin Y.F., Song L.C., Wang C.G., Cui W.H., Zheng Z., Yan D.W., Li Z.J. (2019). NADPH Oxidase Isoforms Are Involved in Glucocorticoid-Induced Preosteoblast Apoptosis. Oxid. Med. Cell Longev..

[3] Ulla A., Uchida T., Miki Y., Sugiura K., Higashitani A., Kobayashi T., Ohno A., Nakao R., Hirasaka K., Sakakibara I. (2021). Morin attenuates dexamethasone-mediated oxidative stress and atrophy in mouse C_2_C1_2_ skeletal myotubes. Arch. Biochem. Biophys..

[4] Seok Y.M., Yoo J.M., Nam Y., Kim J., Kim J.S., Son J.H., Kim H.J. (2021). Mountain ginseng inhibits skeletal muscle atrophy by decreasing muscle RING finger protein-1 and atrogin1 through forkhead box O_3_ in L6 myotubes. J. Ethnopharmacol..

[5] Wang R., Li K., Wang H., Jiao H., Wang X., Zhao J., Lin H. (2019). Endogenous CSE/Hydrogen Sulfide System Regulates the Effects of Glucocorticoids and Insulin on Muscle Protein Synthesis. Oxid. Med. Cell Longev..

[6] Jesus I., Herrera N.A., Andreo J.C., Santos C.F., Amaral S.L. (2020). Training counteracts DEX-induced microvascular rarefaction by improving the balance between apoptotic and angiogenic proteins. Steroids.

[7] Langendorf E.K., Rommens P.M., Drees P., Ritz U. (2021). Dexamethasone Inhibits the Pro-Angiogenic Potential of Primary Human Myoblasts. Int. J. Mol. Sci..

[8] Chazaud B. (2020). Inflammation and skeletal muscle regeneration: Leave it to the macrophages!. Trends Immunol..

[9] Wang Y., Yu R., Wu L., Yang G. (2020). Hydrogen sulfide signaling in regulation of cell behaviors. Nitric Oxide.

[10] Yang R., Jia Q., Li Y., Mehmood S. (2020). Protective effect of exogenous hydrogen sulfide on diaphragm muscle fibrosis in streptozotocin-induced diabetic rats. Exp. Biol. Med..

[11] Lu F., Lu B., Zhang L., Wen J., Wang M., Zhang S., Li Q., Shu F., Sun Y., Liu N. (2020). Hydrogen sulphide ameliorating skeletal muscle atrophy in db/db mice via Muscle RING finger 1 S-sulfhydration. J. Cell. Mol. Med..

[12] Faul F., Erdfelder E., Buchner A., Lang A.G. (2009). Statistical power analyses using G*Power 3.1: Tests for correlation and regression analyses. Behav. Res. Methods.

[13] Desgeorges T., Caratti G., Mounier R., Tuckermann J., Chazaud B. (2019). Glucocorticoids Shape Macrophage Phenotype for Tissue Repair. Front. Immunol..

[14] Kim S., Kim K., Park J., Jun W. (2021). Curcuma longa L. Water Extract Improves Dexamethasone-Induced Sarcopenia by Modulating the Muscle-Related Gene and Oxidative Stress in Mice. Antioxidants.

[15] Takenouchi T., Morozumi T., Wada E., Suzuki S., Nishiyama Y., Sukegawa S., Uenishi H. (2021). Dexamethasone enhances CD163 expression in porcine IPKM immortalized macrophages. Vitro Cell Dev. Biol. Anim..

[16] Baek K.W., Jung Y.K., Kim J.S., Park J.S., Hah Y.S., Kim S.J., Yoo J.I. (2020). Rodent Model of Muscular Atrophy for Sarcopenia Study. J. Bone. Metab..

[17] Anderson L.J., Liu H., Garcia J.M. (2017). Sex Differences in Muscle Wasting. Adv. Exp. Med. Biol..

[18] Karnia M.J., Korewo D., Myslinska D., Ciepielewski Z.M., Puchalska M., Konieczna-Wolska K., Kowalski K., Kaczor J.J. (2021). The Positive Impact of Vitamin D on Glucocorticoid-Dependent Skeletal Muscle Atrophy. Nutrients.

[19] Revenko O., Pavlovskiy Y., Savytska M., Yashchenko A., Kovalyshyn V., Chelpanova I., Varyvoda O., Zayachkivska O. (2021). Hydrogen Sulfide Prevents Mesenteric Adipose Tissue Damage, Endothelial Dysfunction, and Redox Imbalance From High Fructose Diet-Induced Injury in Aged Rats. Front. Pharmacol..

[20] El-Sayed S.S., Shahin R.M., Fahmy A., Elshazly S.M. (2021). Quercetin ameliorated remote myocardial injury induced by renal ischemia/reperfusion in rats: Role of Rho-kinase and hydrogen sulfide. Life Sci..

[21] Zdero R., Borkowski M.M., Coirault C. (2017). Measuring the contraction force, velocity, and length of skeletal muscle. Experimental Methods in Orthopaedic Biomechanics.

[22] Yue L., Talukder M.A.H., Gurjar A., Lee J.I., Noble M., Dirksen R.T., Chakkalakal J., Elfar J.C. (2019). 4-Aminopyridine attenuates muscle atrophy after sciatic nerve crush injury in mice. Muscle Nerve.

[23] Garcia R.A., Vanelli C.P., Pereira O.d.S., Corrêa J.O.d.A. (2018). Comparative analysis for strength serum sodium and potassium in three different methods: Flame photometry, ion-selective electrode (ISE) and colorimetric enzymatic. J. Clin. Lab. Anal..

[24] Gulcin İ. (2020). Antioxidants and antioxidant methods: An updated overview. Arch. Toxicol..

[25] Elhadidy M.G., Elmasry A., Elsayed H.R.H., El-Nablaway M., Hamed S., Elalfy M.M., Rabei M.R. (2021). Modulation of COX-2 and NADPH oxidase-4 by alpha-lipoic acid ameliorates busulfan-induced pulmonary injury in rats. Heliyon.

[26] Lowe R., Shirley N., Bleackley M., Dolan S., Shafee T. (2017). Transcriptomics technologies. PLoS Comput. Biol..

[27] Bisen P.S. (2014). Laboratory Protocols in Applied Life Sciences.

[28] Elsayed H.R.H., Anbar H.S., Rabei M.R., Adel M., El-Gamal R. (2021). Eicosapentaenoic and docosahexaenoic acids attenuate methotrexate-induced apoptosis and suppression of splenic T, B-Lymphocytes and macrophages with modulation of expression of CD3, CD20 and CD68. Tissue Cell.

[29] Schneider C.A., Rasband W.S., Eliceiri K.W. (2012). NIH Image to ImageJ: 25 years of image analysis. Nat. Methods.

[30] Schindelin J., Arganda-Carreras I., Frise E., Kaynig V., Longair M., Pietzsch T., Preibisch S., Rueden C., Saalfeld S., Schmid B. (2012). Fiji: An open-source platform for biological-image analysis. Nat. Methods.

[31] Yamamoto D., Maki T., Herningtyas E.H., Ikeshita N., Shibahara H., Sugiyama Y., Nakanishi S., Iida K., Iguchi G., Takahashi Y. (2010). Branched-chain amino acids protect against dexamethasone-induced soleus muscle atrophy in rats. Muscle Nerve Off. J. Am. Assoc. Electrodiagn. Med..

[32] Sun H., Gong Y., Qiu J., Chen Y., Ding F., Zhao Q. (2014). TRAF6 inhibition rescues dexamethasone-induced muscle atrophy. Int. J. Mol. Sci..

[33] Noh K.K., Chung K.W., Choi Y.J., Park M.H., Jang E.J., Park C.H., Yoon C., Kim N.D., Kim M.K., Chung H.Y. (2014). β–Hydroxy β–Methylbutyrate improves dexamethasone-induced muscle atrophy by modulating the muscle degradation pathway in SD rat. PLoS ONE.

[34] Canepari M., Agoni V., Brocca L., Ghigo E., Gnesi M., Minetto M., Bottinelli R. (2018). Structural and molecular adaptations to dexamethasone and unacylated ghrelin administration in skeletal muscle of the mice. J. Physiol. Pharmacol..

[35] Baehr L.M., Furlow J.D., Bodine S.C. (2011). Muscle sparing in muscle RING finger 1 null mice: Response to synthetic glucocorticoids. J. Physiol..

[36] Yamada T., Ashida Y., Tatebayashi D., Himori K. (2019). Myofibrillar function differs markedly between denervated and dexamethasone-treated rat skeletal muscles: Role of mechanical load. PLoS ONE.

[37] Dunlap K.R., Steiner J.L., Rossetti M.L., Kimball S.R., Gordon B.S. (2021). A clinically relevant decrease in contractile force differentially regulates control of glucocorticoid receptor translocation in mouse skeletal muscle. J. Appl. Physiol..

[38] Loureiro A.C.C., Rêgo-Monteiro I.C.d., Louzada R.A., Ortenzi V.H., Aguiar A.P.d., Abreu E.S.d., Cavalcanti-de-Albuquerque J.P.A., Hecht F., Oliveira A.C.d., Ceccatto V.M. (2016). Differential expression of NADPH oxidases depends on skeletal muscle fiber type in rats. Oxidative Med. Cell. Longev..

[39] Osório Alves J., Matta Pereira L., Cabral Coutinho do Rêgo Monteiro I., Pontes dos Santos L.H., Soares Marreiros Ferraz A., Carneiro Loureiro A.C., Calado Lima C., Leal-Cardoso J.H., Pires Carvalho D., Soares Fortunato R. (2020). Strenuous acute exercise induces slow and fast twitch-dependent NADPH oxidase expression in rat skeletal muscle. Antioxidants.

[40] Mostafa A.F., Samir S.M. (2019). Ferulic Acid Promotes Growth of Both Fast Glycolytic and Slow Oxidative Skeletal Muscles in Corticosteroid-Induced Rat Myopathy. Med. J. Cairo Univ..

[41] Bitar M.S., Nader J., Al-Ali W., Al Madhoun A., Arefanian H., Al-Mulla F. (2018). Hydrogen Sulfide Donor NaHS Improves Metabolism and Reduces Muscle Atrophy in Type 2 Diabetes: Implication for Understanding Sarcopenic Pathophysiology. Oxid. Med. Cell Longev..

[42] Ichinoseki-Sekine N., Smuder A.J., Morton A.B., Hinkley J.M., Mor Huertas A., Powers S.K. (2021). Hydrogen sulfide donor protects against mechanical ventilation-induced atrophy and contractile dysfunction in the rat diaphragm. Clin. Transl. Sci..

[43] Wang X.L., Pan L.L., Long F., Wu W.J., Yan D., Xu P., Liu S.Y., Qin M., Jia W.W., Liu X.H. (2018). Endogenous Hydrogen Sulfide Ameliorates NOX4 Induced Oxidative Stress in LPS-Stimulated Macrophages and Mice. Cell Physiol. Biochem..

[44] Yi J., Yuan Y., Zheng J., Zhao T. (2019). Hydrogen sulfide alleviates uranium-induced rat hepatocyte cytotoxicity via inhibiting Nox4/ROS/p38 MAPK pathway. J. Biochem. Mol. Toxicol..

[45] Scheid S., Goeller M., Baar W., Wollborn J., Buerkle H., Schlunck G., Lagreze W., Goebel U., Ulbrich F. (2021). Hydrogen Sulfide Reduces Ischemia and Reperfusion Injury in Neuronal Cells in a Dose- and Time-Dependent Manner. Int. J. Mol. Sci..

[46] Tang J.J., Podratz J.L., Lange M., Scrable H.J., Jang M.-H., Windebank A.J. (2017). Mechano growth factor, a splice variant of IGF-1, promotes neurogenesis in the aging mouse brain. Mol. Brain.

[47] Jing X., Ye Y., Bao Y., Zhang J., Huang J., Wang R., Guo J., Guo F. (2018). Mechano-growth factor protects against mechanical overload induced damage and promotes migration of growth plate chondrocytes through RhoA/YAP pathway. Exp. Cell Res..

[48] Abdelmonem M., Shahin N.N., Rashed L.A., Amin H.A.A., Shamaa A.A., Shaheen A.A. (2019). Hydrogen sulfide enhances the effectiveness of mesenchymal stem cell therapy in rats with heart failure: In vitro preconditioning versus in vivo co-delivery. Biomed. Pharmacother..

[49] Sha Y., Yang L., Lv Y. (2019). MGF E peptide improves anterior cruciate ligament repair by inhibiting hypoxia-induced cell apoptosis and accelerating angiogenesis. J. Cell. Physiol..

[50] Cheng Z., Kishore R. (2020). Potential role of hydrogen sulfide in diabetes-impaired angiogenesis and ischemic tissue repair. Redox Biol..

[51] Zhao L., Liu X., Zhang J., Dong G., Xiao W., Xu X. (2020). Hydrogen Sulfide Alleviates Skeletal Muscle Fibrosis via Attenuating Inflammation and Oxidative Stress. Front. Physiol..

[52] di Villa Bianca R.d.E., Mitidieri E., Donnarumma E., Tramontano T., Brancaleone V., Cirino G., Bucci M., Sorrentino R. (2015). Hydrogen sulfide is involved in dexamethasone-induced hypertension in rat. Nitric Oxide.

[53] Micheli L., Mitidieri E., Turnaturi C., Vanacore D., Ciampi C., Lucarini E., Cirino G., Ghelardini C., Sorrentino R., Di Cesare Mannelli L. (2022). Beneficial Effect of H2S-Releasing Molecules in an In Vitro Model of Sarcopenia: Relevance of Glucoraphanin. Int. J. Mol. Sci..

